# miR-218 suppresses tumor growth and enhances the chemosensitivity of esophageal squamous cell carcinoma to cisplatin

**DOI:** 10.3892/or.2022.8265

**Published:** 2022-01-17

**Authors:** Hang Tian, Lei Hou, Yu-Mei Xiong, Jun-Xiang Huang, Ying-Jun She, Xiao-Bao Bi, Xing-Rong Song

Oncol Rep 33: 981-989, 2015; DOI: 10.3892/or.2014.3657

Following the publication of this paper, it was drawn to the Editors attention by a concerned reader that certain of the western blotting data shown in Fig. 6 and the tumor images shown in Fig. 7A were strikingly similar to data appearing in different form in other articles by different authors. Owing to the fact that the contentious data in the above article had already been published elsewhere, or were already under consideration for publication, prior to its submission to *Oncology Reports*, the Editor has decided that this paper should be retracted from the Journal. After having been in contact with the authors, they agreed with the decision to retract the paper. The Editor apologizes to the readership for any inconvenience caused.

